# *MiR-20a-5p* functions as a potent tumor suppressor by targeting *PPP6C* in acute myeloid leukemia

**DOI:** 10.1371/journal.pone.0256995

**Published:** 2021-09-29

**Authors:** Fengchang Bao, Lei Zhang, Xiaohang Pei, Cheng Lian, Yanhui Liu, Hongna Tan, Pingchong Lei

**Affiliations:** 1 Department of Hematology, Henan Provincial People’s Hospital, Zhengzhou, Henan, China; 2 Blood institute, Henan Provincial People’s Hospital, Zhengzhou, Henan, China; 3 Medical Imaging Center, Henan Provincial People’s Hospital, Zhengzhou, Henan, China; Indian Institute of Technology Delhi, INDIA

## Abstract

Acute myeloid leukemia (AML) is as a highly aggressive and heterogeneous hematological malignancy. *MiR-20a-5p* has been reported to function as an oncogene or tumor suppressor in several tumors, but the clinical significance and regulatory mechanisms of *miR-20a-5p* in AML cells have not been fully understood. In this study, we found *miR-20a-5p* was significantly decreased in bone marrow from AML patients, compared with that in healthy controls. Moreover, decreased *miR-20a-5p* expression was correlated with risk status and poor survival prognosis in AML patients. Overexpression of *miR-20a-5p* suppressed cell proliferation, induced cell cycle G0/G1 phase arrest and apoptosis in two AML cell lines (THP-1 and U937) using CCK-8 assay and flow cytometry analysis. Moreover, *miR-20a-5p* overexpression attenuated tumor growth in vivo by performing tumor xenograft experiments. Luciferase reporter assay and western blot demonstrated that protein phosphatase 6 catalytic subunit (*PPP6C*) as a target gene of *miR-20a-5p* was negatively regulated by *miR-20a-5p* in AML cells. Furthermore, *PPP6C* knockdown imitated, while overexpression reversed the effects of *miR-20a-5p* overexpression on AML cell proliferation, cell cycle G1/S transition and apoptosis. Taken together, our findings demonstrate that *miR-20a-5p*/*PPP6C* represent a new therapeutic target for AML and a potential diagnostic marker for AML therapy.

## Introduction

Acute myeloid leukemia (AML), as a highly aggressive and heterogeneous hematological malignancy, is characterized by abnormal growth of bone marrow stromal cells and undifferentiated myeloid progenitor cells in the bone marrow and peripheral blood [1, 2]. At present, the principal therapeutic approaches, including chemotherapy, targeted therapy, and hematopoietic stem cell transplantation, have greatly improved the therapeutic outcomes of patients with AML [3, 4]. Unfortunately, the prognosis and outcomes remain still poor in AML patients at advanced stage, which might be correlated with major problems, such as serious infection and bleeding [5–7]. Therefore, it is of great importance to elucidate the molecular mechanisms responsible for the progression of AML.

MicroRNAs (mRNAs/miRs) are small noncoding RNA molecules consisting of 18–24 nucleotides that function as post-transcriptional regulator on gene expression by targeting mRNAs via binding to their 3′-untranslated region (3′-UTR) [8]. Increasing evidence suggests that miRNAs are frequently aberrantly expressed in malignancies and function as tumor suppressors or oncogenes by participating in diverse biological functions, including cell proliferation, apoptosis and differentiation [9, 10]. In recent years, *miR-20a-5p*, a 23-nucleotides-length non-coding RNA, has been reported to be involved in carcinogenesis. For instance, Bai et al [11] showed that *miR-20a-5p* was highly expressed in triple-negative breast cancer and promoted the cell proliferation through targeting runt-related transcription factor 3 (*RUNX3*). *MiR-20a-5p* overexpression contributed to hepatocellular carcinoma (HCC) cell proliferation and migration through reducing the translation of *RUNX3* [12]. Similarly, Huang et al [13] demonstrated that *miR-20a-5p* promoted radio-resistance in nasopharyngeal cancer cells via repression of *Rab27B*. In addition to act as an oncogene, the suppressive role of *miR-20a-5p* was also manifested in endometrial cancer cells by targeting *STAT3* [14], neuroblastoma cells by targeting *ATG7* [15], as well as multi-drug resistance in osteosarcoma by cells targeting *KIF26B* [16] and *SDC2* [17]. What interests us most is that a recent study by Ping et al [18] reported that knockdown of *circ_0009910* inhibited AML cell proliferation and induced apoptosis through sponging *miR-20a-5p*. Nevertheless, the clinical significance and regulatory mechanisms of *miR-20a-5p* in AML cells have not been fully understood and needs to be further explored.

Protein phosphatase 6 catalytic subunit (*PPP6C/PP6C*) is a human ortholog of the yeast protein Sit4 and conserved phosphatase among eukaryotes from yeast to humans, which has been shown to regulate mitotic spindle formation by dephosphorylating Aurora A bound to its activator *TPX2* and be required for normal cell cycle G1 to S transition [19]. PPP6C protein expression was significantly increased in glioma tissues [20]. Similarly, Zhu et al [21] reported that *PPP6C* expression was increased and directly regulated by miR-335 in cervical cancer. Functionally, loss of *PPP6C* in mouse keratinocytes increases susceptibility to ultraviolet-B-induced carcinogenesis [22]. NF-κB activation enhances keratinocyte proliferation by inhibiting *PPP6C* expression directly through the induction of miR-31 [23]. Our online bioinformatic prediction identified that *PPP6C* as a target gene of *miR-20a-5p*. However, the functional role of *PPP6C* and *miR-20a-5p/PPP6C* axis in AML cell proliferation have not been investigated.

In the present study, we first determined the expression of *miR-20a-5p* in bone marrow from AML patients and healthy controls. The correlation between *miR-20a-5p* and clinicopathological variables and survival prognosis was analyzed in AML patients. The in vitro experiments, including CCK-8 assay and flow cytometry and in vivo tumor xenograft experiments were performed to investigate the role of *miR-20a-5p* on AML tumorigenesis. Moreover, the association between *miR-20a-5p* and *PPP6C* was validated in AML cells.

## Materials and methods

### Patients and specimen collection

All specimens were collected through bone marrow aspiration from patients diagnosed as AML (n = 61) and age matched healthy volunteers (n = 61) in the Henan Provincial People’s Hospital (Henan, China) between September 2017 and October 2019. None of the patients received anti-tumor therapies, including chemotherapy or radiotherapy before sample collection. All obtained specimens were immediately kept at −80°C until further analysis. Some basic clinicopathological variables of AML patients with survival information were recorded. All patients were followed-up every 2–4 months for at least 5 years and the overall survival was defined as the period of time from diagnosis to death. This study was performed in accordance with the declaration of Helsinki and Ethic Committee of Henan Provincial People’s Hospital.

### Cell culture and transfection

Four AML cell lines (Kasumi-1, THP-1, U937 and HL-60) and a normal human bone marrow stromal cell line HS-5 were provided by American Type Culture Collection (ATCC, Manassas, VA, USA). All cell lines were cultured in RPMI-1640 medium (Gibco, CA, USA) with 10% FBS (Gibco) at 37°C in a humidified incubator containing 5% CO_2_. Oligonucleotides, including *miR-20a-5p* mimics, negative control (miR-NC), small interfering RNA targeting *PPP6C* (*si-PPP6C*), si-NC and *PPP6C* overexpression plasmids were purchased from RiboBio Co., Ltd. (Guangzhou, China). THP-1 or U937 cells were seeded into six-well plates and transfected with the above synthetic oligonucleotides for 48 h according to experimental requirements using lipofectamine 3000 (Thermo Fisher Scientific, Waltham, MA, USA).

### RNA isolation and quantitative real time PCR

Total RNA was isolated using Trizol reagent (Invitrogen, Carlsbad, CA, USA). Reverse transcription was performed with SuperScript™ IV Reverse Transcriptase (ThermoFisher Scientific). Quantification of miR-20a-5p was performed using Hairpin‐it™ miRNA qPCR Quantitation Kit (GenePharma, China) with the condition: 95°C for 3 min, and 39 circles of 95°C for 10s and 60°C for 30 s. The specific primers used in this study were as follows: *miR-20a-5p*, forward: 5′-GTAAAGTGCTTATAGTGCAG-3′ and reverse: 5′-GTCGTATCCAGTGCGTGTCG-3′; U6 forward, 5′-CTCGCTTCGGCAGCACA-3′, reverse, 5′-AACGCTTCACGAATTTGCGT-3′. Relative quantification of *miR-20a-5p* expression was achieved using the 2^−ΔΔCt^ method by normalization against endogenous control U6.

### Cell proliferation analysis

Cell proliferation was assessed using Cell Counting Kit-8 assay (Dojindo Laboratories). In brief, approximately 0.5 × 10^4^ transfected cells were seeded in each well of 96-well plates and cultured overnight. After incubation for 24, 48, 72 and 96 h, respectively, cells in each well were incubated with 10 μl CCK-8 reagents for another 1 h before the optical density (OD) value was measured at 450 nm by a microplate reader.

### Cell cycle analysis

For cell cycle analysis, approximately 2 × 10^6^ transfected cells were harvested by trypsin treatment and centrifugation at 300 g for 5 min. After washed with PBS twice, cells were fixed with 75% ethanol overnight at 4°C, rehydrated with PBS at room temperature and stained with 500 μL propidium iodide (PI) solution for 15 min in darkness. Finally, cell cycle distribution was analyzed by a BD FACSCanto II (BD Biosciences, San Jose, CA).

### Apoptosis analysis

After 48 h transfection, cells were collected and seeded into 24-well plates at a density of 2 × 10^5^ cells per well. After washed twice with PBS, cells were resuspended in 500 μl binding buffer containing 10 μl fluorescein isothiocyanate (FITC) Annexin V and 10 μl PI solution (Sigma‐Aldrich) for 10 min, followed by apoptotic analysis through a BD FACSCanto II.

### Luciferase reporter assay

Based on the predicted binding sites between *miR-20a-5p* and *PPP6C* on TargetScan7.1 (http://www.targetscan.org), the sequences of *PPP6C* 3’UTR mRNA containing either wild-type (WT) or mutant (MUT) *miR-20a-5p* binding fragments were synthesized by GeneScript (Nanjing, China), which were then inserted into pmirGLO dual-luciferase miRNA target expression vector (Promega, Madison, WI, USA). Constructed recombinant plasmids, including *PPP6C*-WT or *PPP6C*-MUT were transfected into THP-1 or U937 cells with *miR-20a-5p* mimics or *miR-NC* by lipofectamine 3000 following the manufacturer’s instructions. After 48 h, luciferase activity was measured using a Dual Luciferase Reporter Assay system (Promega). Relative luciferase activities were calculated by normalizing Firefly luciferase activity to Renilla luciferase activity.

### Western blot analysis

Total protein sample was extracted using a RIPA lysis kit (Beyotime, Shanghai, China) and protein concentration was determined by BCA protein assay (Beyotime). Approximately 30 μg of protein sample was separated on 12% sodium dodecyl sulphate polyacrylamide gel electrophoresis (SDS‐PAGE) and transferred onto PVDF membranes (Millipore, Bedford, MA, USA). Block of membranes was performed with 5% nonfat milk in Tris-buffered saline containing 0.1% Tween-20 (TBST) for 2 h, the membranes were incubated with primary antibodies against PPP6C and GAPDH (Abcam, Cambridge, MA, USA) overnight at 4°C, followed by incubation with horseradish peroxidase-conjugated secondary antibody. Immunoreactive bands were visualized with an enhanced chemiluminescence kit (Millipore).

### Tumor xenograft experiments

Four‐week‐old male BALB/c nude mice were purchased from the Animal Center of Nanjing Medical University and kept in specific pathogen-free cages with standard rodent chow. A total of 3 × 10^6^ *miR-20a-5p* mimics or miR-NC transfected THP-1 cells in 200 μl PBS were subcutaneously injected into the right posterior flank of equal nude mice. The tumor size was measured at 3, 7, 14 and 28 day, respectively by a caliper after cell implantation. Accordingly, tumor volume was calculated by the formula: volume (cm^3^)  =  (length × width ^2^)/2. On 28 day, the mice were sacrificed and tumor tissues were removed for weighing and *miR-20a-5p* expression analysis. All procedures of animal experiments were conducted in accordance with the Declaration of Helsinki and approved by the Animal Care Committee of the Xi’an Jiaotong University.

### Statistical analysis

After statistical analysis by GraphPad Prism (Version 6.0), the quantitative data were expressed as mean ± SD from three independent experiments. The association of *miR-20a-5p* expression with clinicopathological variables of patients with AML was analyzed by Chi-square test. The overall survival rate estimates survival time were calculated using the Kaplan-Meier method with log-rank test. The differences among two groups were estimated by Student’s t-test or one-way ANOVA with Tukey post hoc test for multiple groups. The values of *p* less than 0.05 were considered to be statistically significant.

## Results

### *MiR-20a-5p* was downregulated and correlated with worse prognosis in AML patients

**Fig 1:**
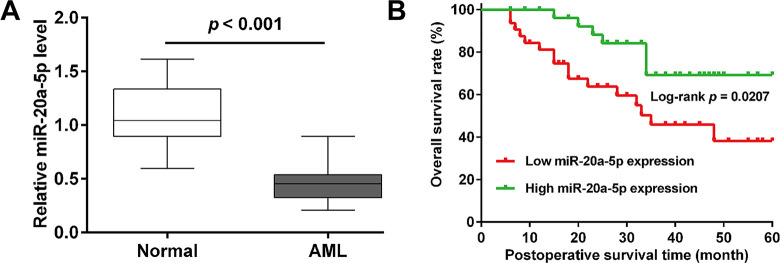
The expression and prognosis of *miR-20a-5p* in AML patients. (A) Expression of *miR‐20a-5p* in bone marrow from AML (n = 61) and age matched healthy volunteers (n = 61) were detected by quantitative real time PCR analysis. (B) Kaplan–Meier analysis of overall survival rate of AML patients with low or high *miR-20a-5p* expression.

To explore the potential role of *miR-20a-5p* in AML development, the expression of *miR-20a-5p* was first analyzed by quantitative real time PCR in bone marrow from 61 AML patients and healthy controls. As shown in **Fig 1A**, *miR-20a-5p* expression was significantly downregulated in AML patients compared with matched healthy volunteers. Next, all AML patients were allocated to high and low *miR-20a-5p* expression group based on the median value, which were applied to research the correlation between *miR-20a-5p* expression and clinical features. As illustrated in **Table 1**, *miR-20a-5p* expression was associated with risk status, but not correlated with age, gender, FAB subtypes and relapse. Moreover, we further analyzed the relationship between *miR-20a-5p* levels and survival time in AML patients. The Kaplan-Meier survival cure showed that the patients with low *miR-20a-5p* expression had shorter overall survival rate than those with high *miR-20a-5p* expression (**Fig 1B**). These data indicated that decreased *miR-20a-5p* expression might predict worse prognosis in AML patients.

**Table 1:** The association between miR-20a-5p expression levels and clinicopathological variables of AML patients.

Variable	miR-20a-5p		*P*-values
	Low expression (n = 32)	High expression (n = 29)	(chi-square test)
**Age** (year)			0.088
< 50	14	19	
≥ 50	18	10	
**Gender**			0.104
Male	22	14	
Female	10	15	
**Risk status**			0.030*
Poor/Intermediate	25	15	
Better	7	14	
**FAB subtypes**			0.536
M1	5	4	
M2	8	6	
M3	10	8	
M4	7	6	
M5	2	5	
**Relapse**			0.621
Yes	19	19	
No	13	10	

Note

*p < 0.05. **Abbreviations:** AML, acute myeloid leukemia; FAB, French-American-British classification

### *MiR-20a-5p* overexpression suppressed cell proliferation, induced G0/G1 phase arrest and apoptosis in AML cells

**Fig 2:**
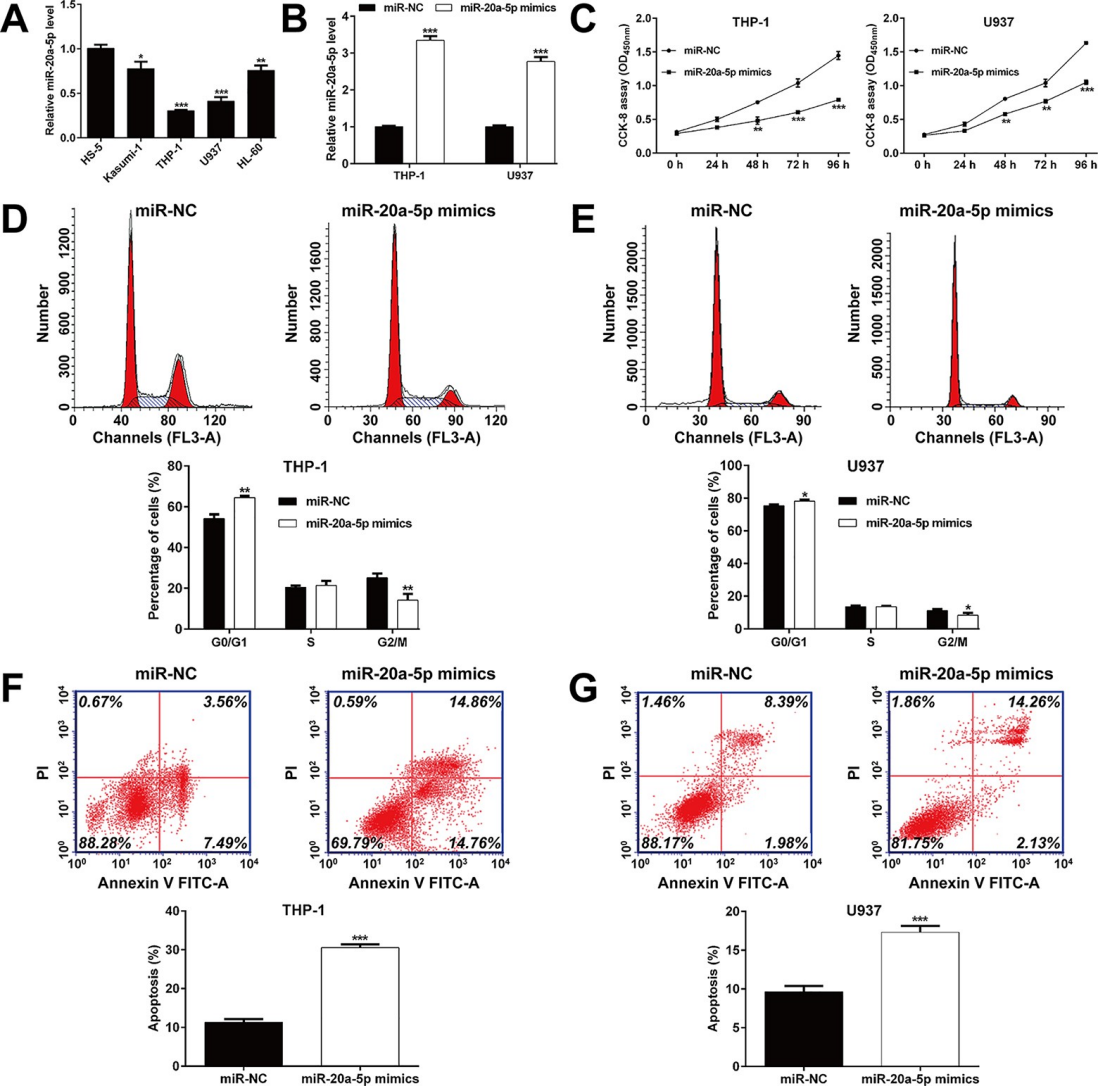
Effects of *miR-20a-5p* overexpression on cell proliferation, cell cycle progression and apoptosis in AML cells. (A) Expression of *miR‐20a-5p* in four AML cell lines (Kasumi-1, THP-1, U937 and HL-60) and a normal human bone marrow stromal cell line HS-5 were detected by quantitative real time PCR analysis. (B) Expression of *miR‐20a-5p* was determined in THP-1 and U937 cells after transfection with *miR-20a-5p* mimics or miR-NC. (C) CCK-8 assay was performed to assess the proliferation of transfected THP-1 and U937 cells. (D-E) The percentage of cells at G0/G1, S and G2/M phase was analyzed by flow cytometry in transfected THP-1 and U937 cells. (F-G) Apoptotic cells were quantified in transfected THP-1 and U937 cells by flow cytometry analysis. Data are presented as mean ± SD of three independent experiments. *p < 0.05, **p < 0.01, ***p < 0.001, compared with miR-NC.

Consistently, we found that *miR-20a-5p* was significantly decreased in four AML cell lines (Kasumi-1, THP-1, U937 and HL-60) compared with a normal human bone marrow stromal cell line HS-5 (**Fig 2A**). Then, two AML cell lines (THP-1 and U937) were selected for gain-of-function assays, which owned lowest *miR-20a-5p* expression level among four AML cell lines, demonstrating better typicality and representation. Quantitative real time PCR analysis confirmed that the expression of *miR-20a-5p* was significantly elevated in THP-1 and U937 cells after *miR-20a-5p* mimics transfection compared with miR-NC transfection (**Fig 2B**). Results of CCK‐8 showed that upregulated *miR-20a-5p* inhibited the proliferation of THP-1 and U937 cells at 48, 72 and 96 h, respectively (**Fig 2C**). Flow cytometry was utilized to further analyze the effects of upregulated *miR-20a-5p* on cell cycle progression and apoptosis status. Cell cycle analysis showed that overexpression of *miR-20a-5p* presented a significant increase in the percentage of cells at G0/G1 phase while a significant decrease in the percentage of cells at G2/M phase in THP-1 (**Fig 2D**) and U937 (**Fig 2E**) cells, which indicated that cell cycle G0/G1 phase arrest was induced by *miR-20a-5p* overexpression. In addition, apoptosis analysis showed that *miR-20a-5p* mimics transfection remarkably promoted cell apoptosis in both THP-1 (**Fig 2F**) and U937 (**Fig 2G**) cells.

### *MiR-20a-5p* negatively regulated *PPP6C* expression by targeting its 3’UTR

**Fig 3:**
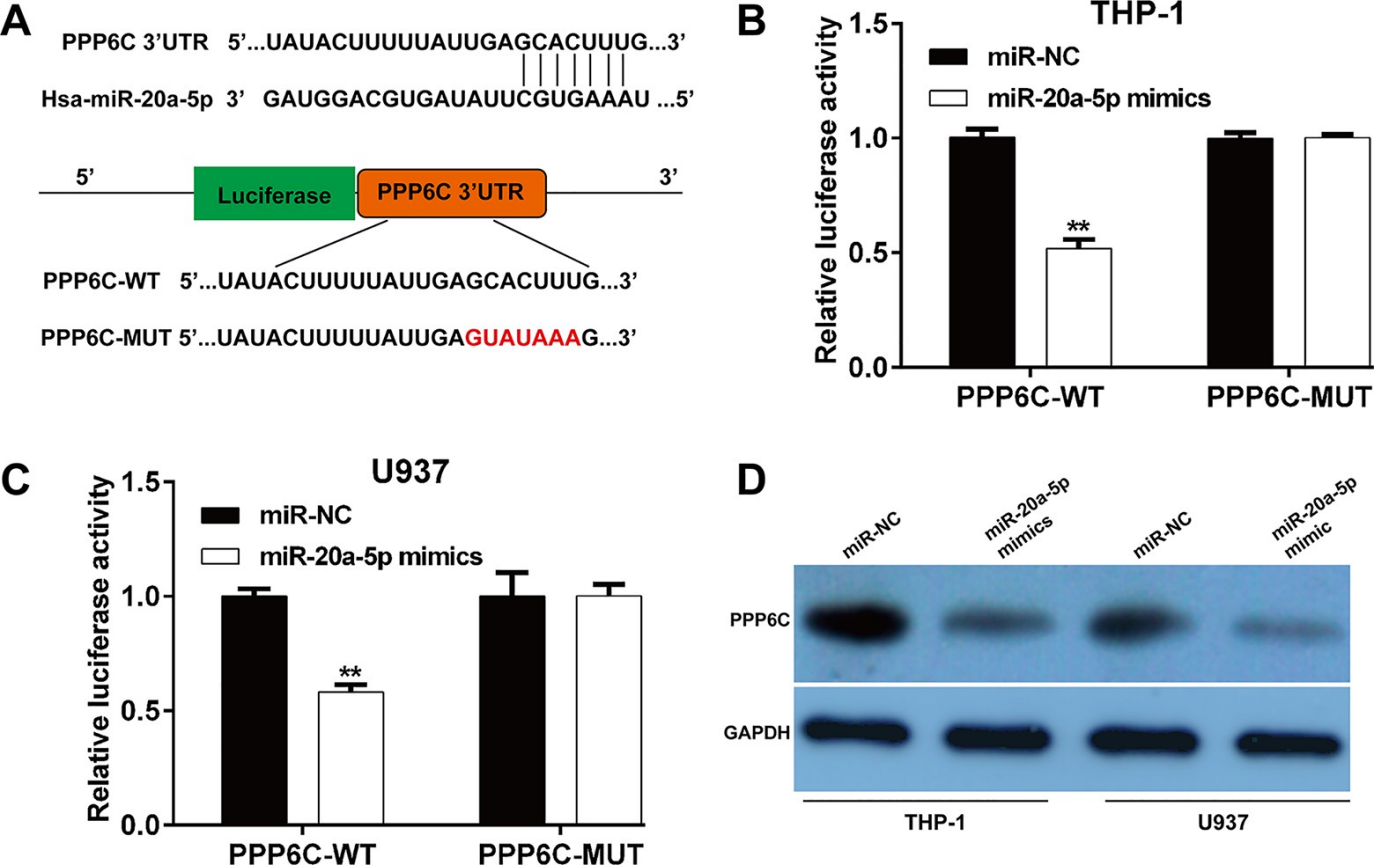
*MiR-20a-5p* negatively regulated *PPP6C* expression by targeting its 3’UTR. (A) The complementary sequences between wild-type or mutant human *PPP6C* 3’-UTRs mRNA and human *miR-20a-5p*. (B) THP-1 and (C) U937 cells were co-transfected with *miR-20a-5p* mimics or miR-NC and the luciferase reporter construct containing the wild-type or mutant *PPP6C* 3′-UTR. For each experiment, the results were normalized to the luciferase activity detected in the cells transfected with the control vector. Data are presented as mean ± SD of three independent experiments. **p < 0.01, compared with miR-NC; (D) The expression of PPP6C protein was detected in THP-1 and U937 cells after transfection with *miR-20a-5p* mimics or miR-NC.

The online public bioinformatics tool TargetScan was used to predict the potential target of *miR-20a-5p*. Among these predicted target genes, *PPP6C*, associated with cell cycle and tumor formation/progression, was selected as a potential target of *miR-20a-5p* for further analysis. To test the effect of *miR-20a-5p* on *PPP6C*, WT and MUT *PPP6C*-3’UTR sequences were cloned into reporter plasmids (**Fig 3A**). The results of luciferase reporter assay showed that ectopic *miR-20a-5p* expression significantly decreased the luciferase activities of *PPP6C*-WT in THP-1 (**Fig 3B**) and U937 (**Fig 3C**) cells. Western blot analysis further demonstrated that *miR-20a-5p* mimics transfection obviously downregulated the expression of *PPP6C* protein in THP-1 and U937 cells compared with miR-NC transfection (**Fig 3D**). These data suggested that *miR-20a-5p* mediated *PPP6C* expression by directing targeting its 3’UTR, which may be involved in the suppressive role in AML.

### Knockdown of *PPP6C* suppressed cell proliferation, induced G0/G1 phase arrest and apoptosis in AML cells

**Fig 4:**
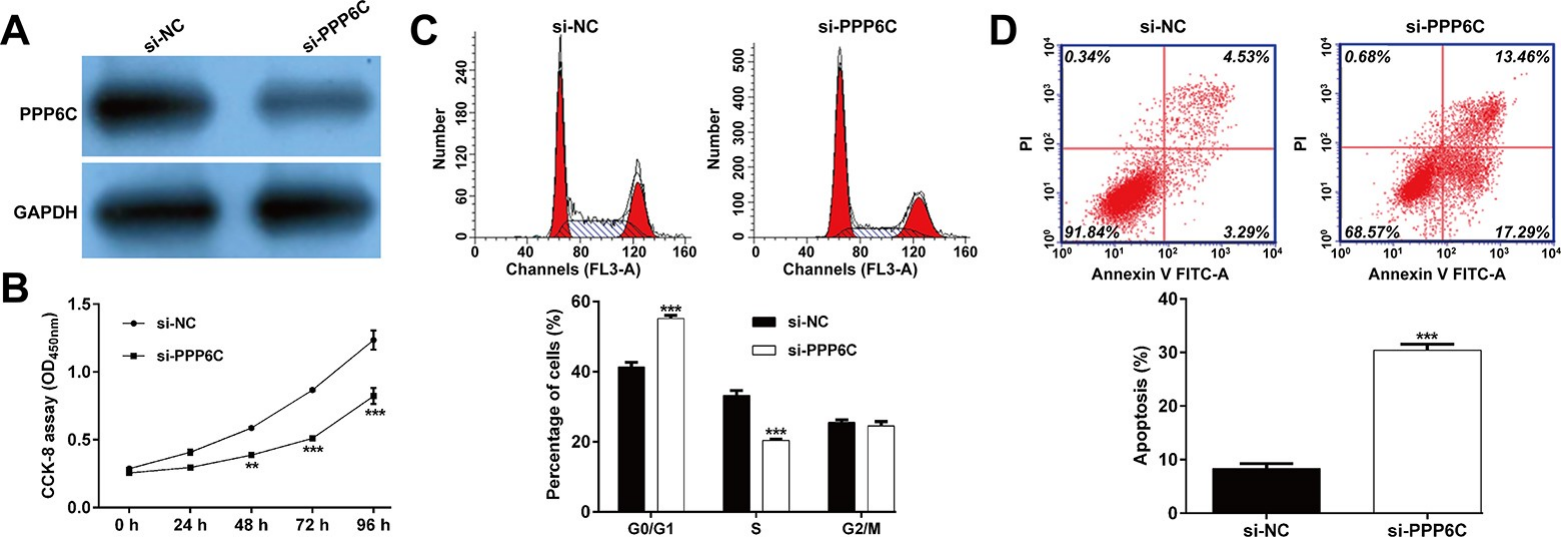
Effects of *PPP6C* knockdown on cell proliferation, cell cycle progression and apoptosis in AML cells. THP-1 cells were transfected with si-PPP6C or si-NC, respectively. (A) Western blot analysis was performed to measure the protein level of PPP6C in transfected THP-1 cells. (B) Cell proliferation was assessed by CCK-8 assay in transfected THP-1 cells. Flow cytometry assay was applied to determined cell cycle distribution (C) and apoptotic rate (D) in transfected THP-1 cells. Data are presented as mean ± SD of three independent experiments. **p < 0.01, ***p < 0.001, compared with si-NC.

Since *PPP6C* was negatively regulated by *miR-20a-5p* in AML cells, we then performed loss-of-function assay in AML cells by transfection with si-PPP6C or si-NC into THP-1 cells. As shown in **Fig 4A**, the protein level of PPP6C was obviously downregulated in THP-1 cells after si-PPP6C transfection. Consistent with the effects of *miR-20a-5p* in AML cells, we observed that knockdown of *PPP6C* markedly suppressed cell proliferation (**Fig 4B**), induced cell G0/G1 phase arrest (**Fig 4C**) and promoted apoptosis (**Fig 4D**) in THP-1 cells. These data indicated that *PPP6C* played a positive role in AML cell growth and proliferation.

### *PPP6C* overexpression reversed the effects of *miR-20a-5p* on AML cell proliferation, G1/S transition and apoptosis

**Fig 5:**
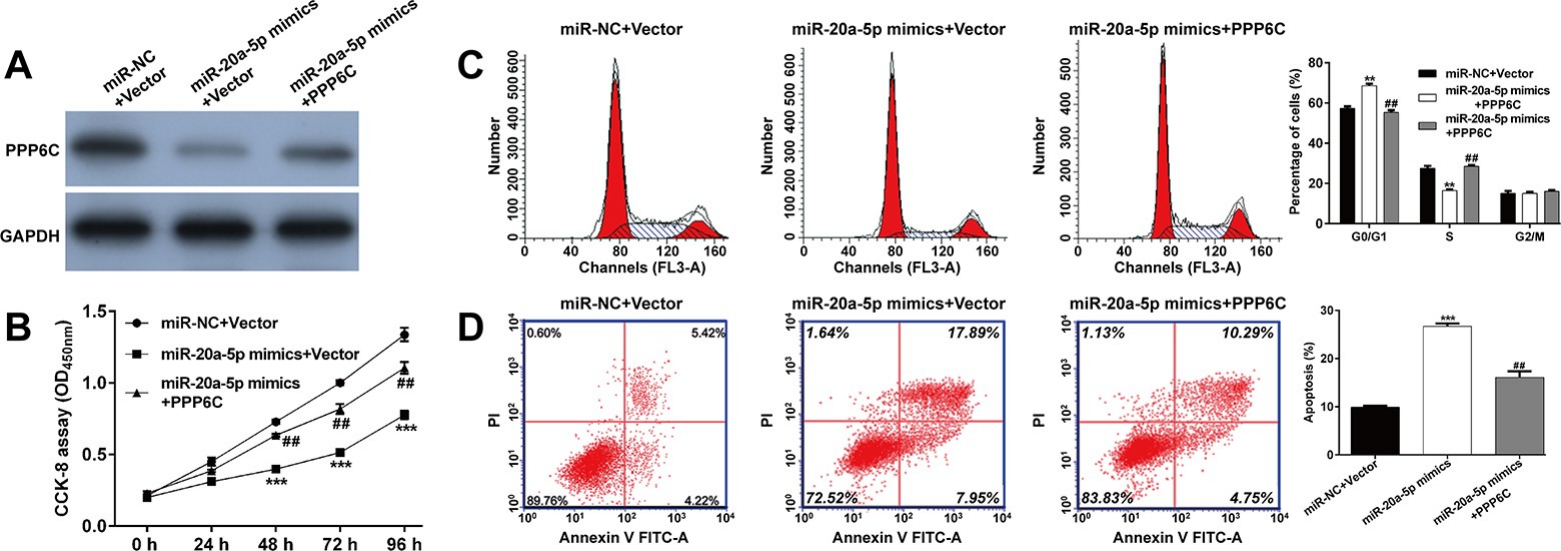
*PPP6C* overexpression reversed the effects of *miR-20a-5p* on AML cell proliferation, G1/S transition and apoptosis. THP-1 cells were co-transfected with *miR-20a-5p* mimics and *PPP6C* or vector. (A) The expression of PPP6C protein was detected in transfected THP-1 cells. (B) CCK-8 assay was performed to assess the proliferation of transfected THP-1 cells. (C) Flow cytometry with PI staining was applied to analyze cell cycle distribution in transfected THP-1 cells. (D) Apoptotic rate was determined in transfected THP-1 cells. Data are presented as mean ± SD of three independent experiments. **p < 0.01, ***p < 0.001, compared with miR-NC +Vector; ##*p* < 0.01, compared with *miR-20a-5p* mimics +Vector.

Next, we performed rescue experiments to confirm whether *PPP6C* was the downstream regulator involved in *miR-20a-5p* regulating cell functions in AML cells. THP-1 cells were co-transfected with *miR-20a-5p* mimics and *PPP6C*. As shown in **Fig 5A**, the expression of PPP6C protein was decreased after miR-20a-5p + Vector, compared with miR-NC + Vector transfection, which was recovered after co-transfection with *miR-20a-5p* mimics and *PPP6C*. CCK-8 assay showed that increased *PPP6C* expression partially restored the impaired proliferation induced by *miR-20a-5p* overexpression in THP-1 cells (**Fig 5B**). Moreover, *PPP6C* overexpression reversed the accelerative effects of *miR-20a-5p* on G0/G1 arrest (**Fig 5C**) and apoptosis (**Fig 5D**) in THP-1 cells.

### *MiR-20a-5p* inhibited tumor growth in mice xenograft models

**Fig 6:**
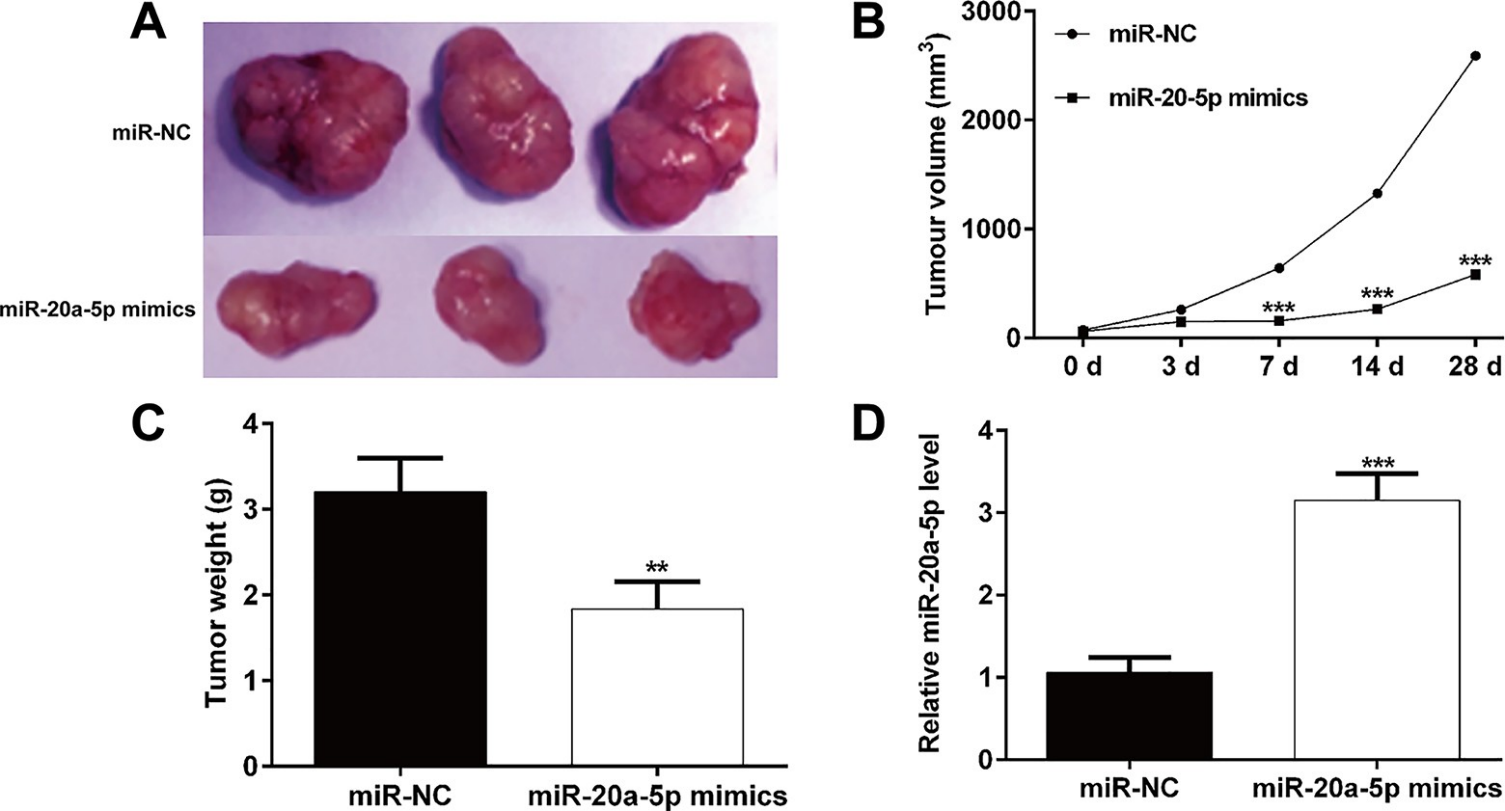
*MiR-20a-5p* inhibited tumorigenicity in vivo. (A) Representative photographs of tumors were obtained from the different groups of nude mice (n = 5 per group) transfected with *miR-20a-5p*‐mimics, and miR‐NC. Tumors were observed by (B) tumor volume and (C) average weight. (D) Expression of *miR-20a-5p* was detected by quantitative real time PCR. Data are presented as mean ± SD of three independent experiments. **p < 0.01, ***p < 0.001, compared with miR-NC.

To reveal the *in vivo* tumor suppressive effects of *miR-20a-5p*, THP-1 cells transfected with *miR-20a-5p* mimics or miR-NC were injected into the flanks of nude mice to generate tumors ectopically. As depicted in **Fig 6A**, mice injected with *miR-20a-5p* mimics transfected-THP-1 cells generated smaller tumor size compared with those with miR-NC transfected cells. After injections of *miR-20a-5p* mimics at consecutive 28 days, tumor growth was significantly lower than that following injection of miR-NC (**Fig 6B**). Moreover, the formed tumor weight was significantly decreased in *miR-20a-5p* mimics group, in comparison with miR-NC group (**Fig 6C**). In addition, quantitative real time analysis confirmed that *miR-20a-5p* expression was upregulated in *miR-20a-5p* mimics-transfected tumors when compared with miR-NC-transfected tumors (**Fig 6D**). Taken together, these results clearly demonstrate that *miR-20a-5p* functioned as a tumor suppressor in vivo.

## Discussion

In this study, we found that *miR-20a-5p* was significantly decreased in bone marrow from AML patients, compared with that in healthy controls. Moreover, decreased *miR-20a-5p* expression was correlated with risk status and poor survival prognosis in AML patients. Consistently, the expression level of *miR-20a-5p* was reported to be downregulated along with clinical staging of neuroblastoma progression [15], in drug-resistant pancreatic cancer cells [24], breast tumors [25, 26] and endometrial cancer tissues [14]. Downregulation of miR-20a-5p was correlated with the malignancy and poor prognosis of glioma patients [27]. HCC patients with lower level of *miR-20a-5p* had significantly shorter OS and PFS survivals after surgical resection [28]. Contrary to our results, miR-20a-5p was upregulated in lung squamous cell carcinoma (SCC) tissues and serum exosomes with considerable clinical value in the diagnosis of male lung SCC patients [29]. The upregulation of *miR-20a-5p* was associated with advanced clinical stages of astrocytoma [30]. Notably, the downregulation of *miR-20a-5p* identified by Ping et al [18] confirmed our data on the decreased expression of *miR-20a-5p* and its correlation with worse prognosis in AML patients.

Functionally, we further demonstrated that overexpression of *miR-20a-5p* significantly suppressed cell proliferation, induced cell cycle G0/G1 phase arrest and apoptosis in two AML cell lines (THP-1 and U937), as well as inhibited tumor growth in mice xenograft models. These findings indicated the tumor suppressive role of *miR-20a-5p* in AML progression. In line with our data, *miR-20a-5p* inhibited cell proliferation and promoted apoptosis in SH-SY5Y cells [15]. Forced expression of *miR-20a-5p* counteracted osteosarcoma cell chemoresistance in both cell culture and tumor xenografts in nude mice [16]. Ping et al [18] further confirmed that *miR-20a-5p* inhibitors reversed *circ_0009910* siRNA-mediated cell proliferation inhibition, cell cycle arrest and cell apoptosis in AML5 cells. Different from the study from Ping et al, our study used two AML cell lines (THP-1 and U937) and performed gain-of-function assay to consistently demonstrated the suppressive effects of *miR-20a-5p* in AML cell proliferation and tumor growth. On the contrary, some studies described the oncogenic role of *miR-20a-5p* in other tumor cells, including colorectal cancer [31], HCC [12] and radio-resistance in nasopharyngeal cancer [13]. These differences in the regulatory role of *miR-20a-5p* might be ascribed to different tissue resources, cell types and different culture conditions.

As our best knowledge, miRNAs bind to the 3’-UTR of their target mRNAs, leading to degradation of mRNA or inhibiting mRNA translation. Herein, *PPP6C* was a bioinformatics target of *miR-20a-5p*, and we further validated *miR-20a-5p* directly targeted *PPP6C* and reduced the expression of PPP6C protein. And indeed, *PPP6C* has been demonstrated to be a target gene of several miRNAs, such as *miR-31* [32], *miR-373* [33] and *miR-335* [21]. Our data additionally indicated that *PPP6C* knockdown imitated, while overexpression reversed the effects of *miR-20a-5p* overexpression on AML cell proliferation, cell cycle G1/S transition and apoptosis, which suggested that *miR-20a-5p* regulated AML cell proliferation, G1/S transition and apoptosis via *PPP6C* repression. In fact, PPP6C protein expression was significantly upregulated in glioma [20] and cervical cancer [21]. The pro-survival effects of *PPP6C* have been manifested in ultraviolet-B-induced carcinogenesis [22] and keratinocyte proliferation [23]. By searching the members of the protein serine/threonine phosphatase family, we found *PPP5C* could significantly promote the tumor cell proliferation and survival, including HCC [34], ovarian cancer [35] and prostate cancer [36]. Interestingly, knockdown of *PPP5C* suppressed the proliferation ability, and led to G0/G1 phase arrest, induced cell apoptosis in leukemic cell line U937 cells [37], which is line with our data showed that *PPP6C* promoting AML cell proliferation.

## Conclusions

In summary, we demonstrated that *miR-20a-5p* was significantly downregulated and correlated with worse prognosis in AML patients. *MiR-20a-5p* overexpression impaired AML cell proliferation, induced cell cycle G0/G1 phase arrest, apoptosis in vitro and tumor growth in vitro. Moreover, we confirmed that *miR-20a-5p* negatively regulated *PPP6C* expression by binding to its 3’-UTR and further confirmed that *PPP6C* was the downstream regulator involved in *miR-20a-5p* regulating AML cell functions. The results of this study indicate that the *miR-20a-5p*/*PPP6C* axis closely correlates with the malignant progression of AML, which might be of potential value as novel therapeutic targets for AML treatment.

## Supporting information

S1 Fig (TIF)
