# Supplementary figures and images for: MiR-20a-5p functions as a potent tumor suppressor by targeting PPP6C in acute myeloid leukemia
Source: PLoS One. 2021 Sep 29;16(9):e0256995. doi: 10.1371/journal.pone.0256995 (PMC8480815; doi:10.1371/journal.pone.0256995)

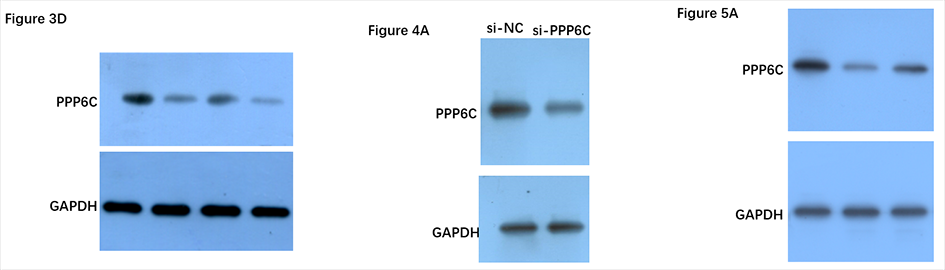

Supplement: S1 Fig — (TIF) [file pone.0256995.s001.tif]
